# Ethephon induced oxidative stress in the olive leaf abscission zone enables development of a selective abscission compound

**DOI:** 10.1186/s12870-017-1035-1

**Published:** 2017-05-16

**Authors:** S. Goldental-Cohen, C. Burstein, I. Biton, S. Ben Sasson, A. Sadeh, Y. Many, A. Doron-Faigenboim, H. Zemach, Y. Mugira, D. Schneider, R. Birger, S. Meir, S. Philosoph-Hadas, V. Irihomovitch, S. Lavee, B. Avidan, G. Ben-Ari

**Affiliations:** 1 Institute of Plant Sciences, ARO, The Volcani Center, 7528809 Rishon LeZion, Israel; 2 The Robert H. Smith Institute of Plant Sciences and Genetics in Agriculture, The Robert H. Smith Faculty of Agriculture, Food and Environment, The Hebrew University of Jerusalem, 76100 Rehovot, Israel; 3 The Agricultural Extension Service of Israel, Bet-Dagan, Israel; 4 Migal – Galilee Technology Center, P.O. Box 831, 11016 Kiryat Shemona, Israel; 5 Agriculture Valley Center, P.O. Box 73, 23100 Migdal Haemeq, Israel

**Keywords:** Abscission, Detachment force, Olive, Mechanical harvesting, Ethylene, Oxidative stress, ROS, Antioxidant

## Abstract

**Background:**

Table olives (*Olea europaea* L.), despite their widespread production, are still harvested manually. The low efficiency of manual harvesting and the rising costs of labor have reduced the profitability of this crop. A selective abscission treatment, inducing abscission of fruits but not leaves, is crucial for the adoption of mechanical harvesting of table olives. In the present work we studied the anatomical and molecular differences between the three abscission zones (AZs) of olive fruits and leaves.

**Results:**

The fruit abscission zone 3 (FAZ3), located between the fruit and the pedicel, was found to be the active AZ in mature fruits and is sensitive to ethephon, whereas FAZ2, between the pedicel and the rachis, is the flower active AZ as well as functioning as the most ethephon induced fruit AZ. We found anatomical differences between the leaf AZ (LAZ) and the two FAZs. Unlike the FAZs, the LAZ is characterized by small cells with less pectin compared to neighboring cells. In an attempt to differentiate between the fruit and leaf AZs, we examined the effect of treating olive-bearing trees with ethephon, an ethylene-releasing compound, with or without antioxidants, on the detachment force (DF) of fruits and leaves 5 days after the treatment. Ethephon treatment enhanced pectinase activity and reduced DF in all the three olive AZs. A transcriptomic analysis of the three olive AZs after ethephon treatment revealed induction of several genes encoding for hormones (ethylene, auxin and ABA), as well as for several cell wall degrading enzymes. However, up-regulation of cellulase genes was found only in the LAZ. Many genes involved in oxidative stress were induced by the ethephon treatment in the LAZ alone. In addition, we found that reactive oxygen species (ROS) mediated abscission in response to ethephon only in leaves. Thus, adding antioxidants such as ascorbic acid or butyric acid to the ethephon inhibited leaf abscission but enhanced fruit abscission.

**Conclusion:**

Our findings suggest that treating olive-bearing trees with a combination of ethephon and antioxidants reduces the detachment force (DF) of fruit without weakening that of the leaves. Hence, this selective abscission treatment may be used in turn to promote mechanized harvest of olives.

**Electronic supplementary material:**

The online version of this article (doi:10.1186/s12870-017-1035-1) contains supplementary material, which is available to authorized users.

## Background

The development of the olive from flower to ripe fruit is a complex process. Flowering usually occurs in April in the north hemisphere and fruit set takes place 2 weeks later. As in many other fruits, olive development follows a double sigmoid pattern until the fruit reaches its full size about mid-September, 150 days post anthesis (DPA). Ripening of the olive is a long, slow process that lasts several months and starts around mid October, 180 DPA. Harvest usually begins in mid-November, 210 DPA [1]. Abscission refers to a set of separation events in which entire plant organs, such as leaves, fruit, or floral organs are shed. During these events, separation usually occurs in specialized, narrow bands of cells termed abscission zones (AZs), which form between the mother plant and each of the above mentioned organs [2]. Most plant species contain one fruit AZ (FAZ); some however, contain more than one. Two FAZs were reported in citrus fruits [3], tomatoes [4], Longkong [5] and others. The peach fruit has three AZs, in which the timing of functional differentiation and the ability to respond to induction by ethylene treatment depend on the fruit developmental stage [6, 7]. Similarly, olive fruit abscission can occur at three different locations: peduncle-branch (FAZ1), pedicel rachis (FAZ2) and fruit-pedicel (FAZ3). Mature olive fruit abscission occurs mainly in FAZ3, to a lesser degree in FAZ2 and rarely in FAZ1 [8]. In olive cultivars ‘Picual’ and ‘Arbequina’, FAZ2 was characterized previously as the immature fruit AZ [9]. In crops, abscission of leaves, flowers, young, and mature fruit are predominantly controlled by contracting actions of ethylene acting as an inducer, and auxin (indole-3-acetic acid, IAA) acting as a suppressor. However, abscisic acid (ABA) is also involved, as ABA levels were shown to increase significantly prior to abscission [10].

Exogenous ethylene induces abscission in most abscising plant systems. In some, but not all cases, endogenous ethylene production increases before abscission. Ethylene interacts with auxin in regulating fruit and leaf abscission by inducing the expression of genes involved in cell separation such as expansins, cellulases and pectinases [11]. Auxin depletion in the AZ is required for AZ cells to respond to ethylene as an abscission signal. This is a general phenomenon that occurs in natural abscission and in abscission induced by various stresses. Stress-induced auxin depletion is mediated by three main intermediate modulators: ethylene, reactive oxygen species (ROS), and carbohydrate starvation [12]. ROS are produced in response to many environmental stresses, such as UV, chilling, salt, water logging, anoxia, dehydration, and pathogen attack [13]. ROS stimulates auxin catabolism [12], and the continuous production of hydrogen peroxide (H_2_O_2_) is involved in leaf abscission signaling [14]. The induction of the apple fruitlet abscission process at the cortex level seems to be orchestrated by a multiple network of interactions between hormones, mainly ABA and ethylene, and other signaling molecules such as ROS [15, 16].

It is evident that abscission is accompanied by a spectrum of changes in proteins including cell wall degrading enzymes [17]. The first enzyme proposed to contribute to wall loosening at the site of abscission was β-1,4-glucanase, or cellulase. The expression of gene family members encoding for this enzyme is upregulated specifically in the AZ after ethylene treatment [18]. Other enzymes involved in the abscission process are polygalacturonases (PGs), whose activity increases during the abscission of different organs. Expansins were associated with cell wall loosening during expansion growth and ripening, and were shown to play a role in cell separation [19]. Transcriptome profiling of the AZ using microarray or RNAseq was characterized in many plant species, such as Arabidopsis [20–22], tomato [23, 24], citrus [25], apple [26], litchi [27], melon [28] and others. The olive fruit AZ transcriptome has been characterized recently comparing changes between the FAZ-rich tissue and fruit at the fruit ripening stage [9], or changes in the FAZ gene expression between table olive harvest stage at 154 days post anthesis (DPA) and full ripening stage at 217 DPA [29]. Gene expression analysis of the mature olive FAZ revealed that sphingolipids are potentially involved in the abscission process of mature olive fruit. Activation of vesicle trafficking involving small GTPases is probably required for cell wall modifications during abscission, and many MYB and bZIP transcription factors are abundantly represented in the fruit AZ, and may regulate downstream processes mostly related to ABA [29].

Olive fruit growth and development lasts for 4–5 months and includes five stages, the last of which is non-climacteric maturation (beginning about 180 days after anthesis). As in other fruit crops, fruit detachment force (DF) declines steadily as fruit matures. This enables harvesting of oil olives when a light green to purple coloration is reached and fruit softening begins. In contrast to this, table olives are harvested towards the end of the mesocarp development stage (before physiological maturation) when DF is very high. This hinders fruit harvesting and increases the effort necessary to carrying out this task.

Currently, table olives are still manually harvested. The major reason for developing mechanical olive harvesting is the shortage of manpower for hand harvesting and increasing labor costs. At present, hand harvesting is the most expensive component of table olive production worldwide [30]. In order to enable mechanical harvest, a selective abscission treatment able to decrease the fruit detachment force (DF) without affecting leaf DF is crucial. Ethylene-releasing compounds such as ethephon (2-chloroethyl phosphonic acid) achieved consistent fruit loosening. However, ethephon application also resulted in attendant leaf loss coincident with fruit loosening [31–33]. The purpose of the present research was to study the three olive AZs at the physiological and molecular levels, in order to develop a differential treatment that will lead to fruit abscission without leaf abscission. For this purpose we characterized the active olive fruit and leaf AZs (LAZ, FAZ2 and FAZ3) in cv. Manzanillo olive trees. FAZ2 was found to be activated by ethylene released by the ethephon treatment. Clear anatomical differences were found between leaf and fruit AZs. In contrast to previous transcriptomic analyses, in this study the modification of gene expression was achieved by ethephon treatment, which induced organ abscission. The transcriptomic analysis revealed that expression of genes in the leaf and fruit AZs responded distinctively to the ethephon stimuli. These differences were manifested in expression of many genes, among them several genes coding for cell wall degrading enzymes and others involved in response to ROS. Antioxidant treatment was shown to repress the ethephon-induced abscission only of leaves. Therefore, our findings suggest that the ethephon-induced abscission mediated via ROS is leaf-specific.

## Methods

### Plant material, treatments and measurements

The work in this study was carried out on olive ‘Manzanillo’ trees selected from our germplasm collection. Our germplasm collection consists of 119, 17 year-old, irrigated olive tree cultivars spaced 5 × 6.5 m apart, located at the Volcani Center (ARO) in Rishon LeZion, Israel [34]. The germplasm collection is in our responsibility and no permissions were necessary to carry out this experiment in accordance with local legislation. Detachment force (DF) measurements of fruits and leaves were performed using a force gauge FG-5000A (Lutron electronic enterprise, Taipei, Taiwan). All experiments and samples collection were performed in September, which is the harvest time of table olives. The percentage of abscised fruits after ethephon treatment was measured by picking the fruit and analyzing the FAZ involved in the fruit abscission. Although we used the term “abscission”, all analyses were carried out on abscised fruits and leaves which were removed by hand.

### Ethephon and antioxidant treatments

Fruiting branches on the tree were dipped in a 2-L container filled with an ethephon agent (pH = 5.5) containing 4% monopotassium phosphate (MPK; Haifa NPK, Haifa, Israel), 0.2% ethrel (Bayer Cropscience, Monheim, Germany) and 0.3% paraffin oil (Drexel, Memphis, TN, USA). The antioxidant treatments were applied by adding either 0.3% ascorbic acid (Galvit C, Biovac, Or Akiva, Israel) or 100 mM butyric acid (Sigma-Aldrich, St. Louis, MO) to the ethephon agent. Control branches were similarly dipped in a 2-L water container. All DF measurements during the experiment were carried out on leaves and fruits while attached to the tree.

### ROS identification

ROS detection was done using the ROS fluorescent probe 2′,7′-dichlorofluorescein diacetate (DCFH-DA; Sigma-Aldrich, St. Louis, MO). The leaf and fruit AZs were excised by hand to approximately 0.1 mm thick vertical sections. Sections were incubated with 25 μM DCFH-DA in 10 mM Tris-HCl (pH 7.4) buffer for 30 min in the dark at 25 °C [35]. The sections were then washed in 10 mM Tris-HCl (pH 7.4) buffer without DCFH-DA, placed on a slide and observed under a confocal laser scanning microscope (Olympus IX81; Olympus, Tokyo, Japan). For each image, 5 different regions were sampled to measure DCF fluorescence intensities which were than background subtracted.

### Pectinase activity assay

The Pectinase activity assay was carried out according to the protocol-online reference (http://www.protocol-online.org/). The leaf and fruit AZs sampled for Pectinase activity assay were excised by hand to an approximate thickness of 1 mm from each side of the AZ region. LAZ, FAZ2 and FAZ3 tissues were ground and dissolved in 40 mM sodium acetate (NaAc; pH = 5). Samples were then centrifuged and the supernatant liquid was incubated with 5% pectin (Sigma-Aldrich, St. Louis, MO) in an equal volume at 40 °C for 1 h. Viscosity of these mixtures was evaluated by taking 1 mL into a glass pipette, and measuring the time it takes the mixture to drain out under gravity, compared to 5% pectin. In order to translate viscosity into pectinase activity we assayed a standard of 5% pectin with 0.2 and 0.4 units of commercial pectinase enzyme (Sigma-Aldrich, St. Louis, MO).

### Anatomical analysis

The leaf and fruit AZs sampled for anatomical analysis were excised by hand to a size of approximately 1 cm^2^ consisting of the AZ region as well as the adjacent tissues. AZ Samples were fixed in formaldehyde: acetic acid: ethanol 70%, 10:5:85, *v*/v (FAA) solution. Fixation was followed by an ethanol dilution series and a subsequent stepwise exchange of ethanol with histoclear. Samples were embedded in paraffin and cut by microtome (Leica RM2245) into sections, then stained with ruthenium red and alcian blue [36]. The stained samples were examined with a light microscope (Olympus BX50) at 5, 10, 20, and 40 magnifications.

### AZ transcriptome profiling

The leaf and fruit AZs sampled for transcriptome profiling were excised by hand to approximately 1 mm thick from each side of the AZ region. Tissues were removed from the tree, immediately excised and frozen in liquid nitrogen. AZ enriched tissues were used to prepare six cDNA libraries from total RNA of the three analyzed AZs (LAZ, FAZ2 and FAZ3) of ‘Manzanillo’ before and 5 days after ethephon treatment, using Illumina’s TruSeq RNA library preparation kit according to the manufacturer’s instructions. Each cDNA library consisted of RNA from about 70 AZs sampled from different fruits or leaves, and was sequenced on Illumina HiSeq 2000. Sequencing was performed by the Sequencing unit of the Technion Genome Center in Haifa, Israel. Raw reads were subjected to a filtering and cleaning procedure using the FASTX Toolkit (http://hannonlab.cshl.edu/fastx_toolkit/ index.html, version 0.0.13.2) for: (1) trimming read-end nucleotides with quality scores <30 using fastq_quality_trimmer; (2) removing reads with less than 70% base pairs with quality score ≤ 30 using fastq_quality_filter. Cleaned reads, obtained after processing, were assembled de novo using Trinity software [37] with trimmomaticSE option to remove adaptors [38]. Only transcripts with a minimum length of 200 bp were analyzed. The transcript quantification (the number of reads per gene) from RNA-Seq data was performed using bowtie2 aligner [39] and the Expectation-Maximization method (RSEM) [40].

### Data analysis

Differential expression analysis was done with edgeR package [41], transcripts that were more than twofold differentially expressed with false discovery- corrected statistical significance of at most 0.01 were considered differentially expressed [42]. Hierarchical clustering of gene expression and visualization of heatmap were performed using R Bioconductor [43], using the log-FPKM values of each gene. For gene ontology (GO) analysis, the de-novo assembled sequences were blasted against the UniRef90 database [44], by which a number of GO annotations were derived for each gene. Singular enrichment analysis (SEA), which lists the enriched GO terms, was performed [Fisher’s Exact Test and false discovery rate (FDR) ≤0.05] based on the five best GO terms of each gene, using the Blast2go interface (https://www.blast2go.com/). Venn diagrams were constructed using the online Venny 2.0 software (http://bioinfogp.cnb.csic.es/tools/venny/).

### Singular enrichment analysis (SEA)

We tested the lists of transcripts regulated in each AZ separately, as well as transcripts regulated uniquely in the LAZ, FAZ2 and FAZ3, as well as transcripts commonly regulated in all three AZs. SEA of each list provided a list of enrichment GO terms for each set of transcripts. SEA generated separated lists for each of the three GO categories: biological processes, molecular functions and cellular components. We focused on the biological process category, assuming it to be the most indicative category in our analysis.

### Statistical analysis

The data collected for fruit and leaf DF and gene expression was subjected to one-way analysis of variance (ANOVA) using JMP software [45].

## Results

### Annual course of leaf and fruit detachment forces

**Fig. 1:**
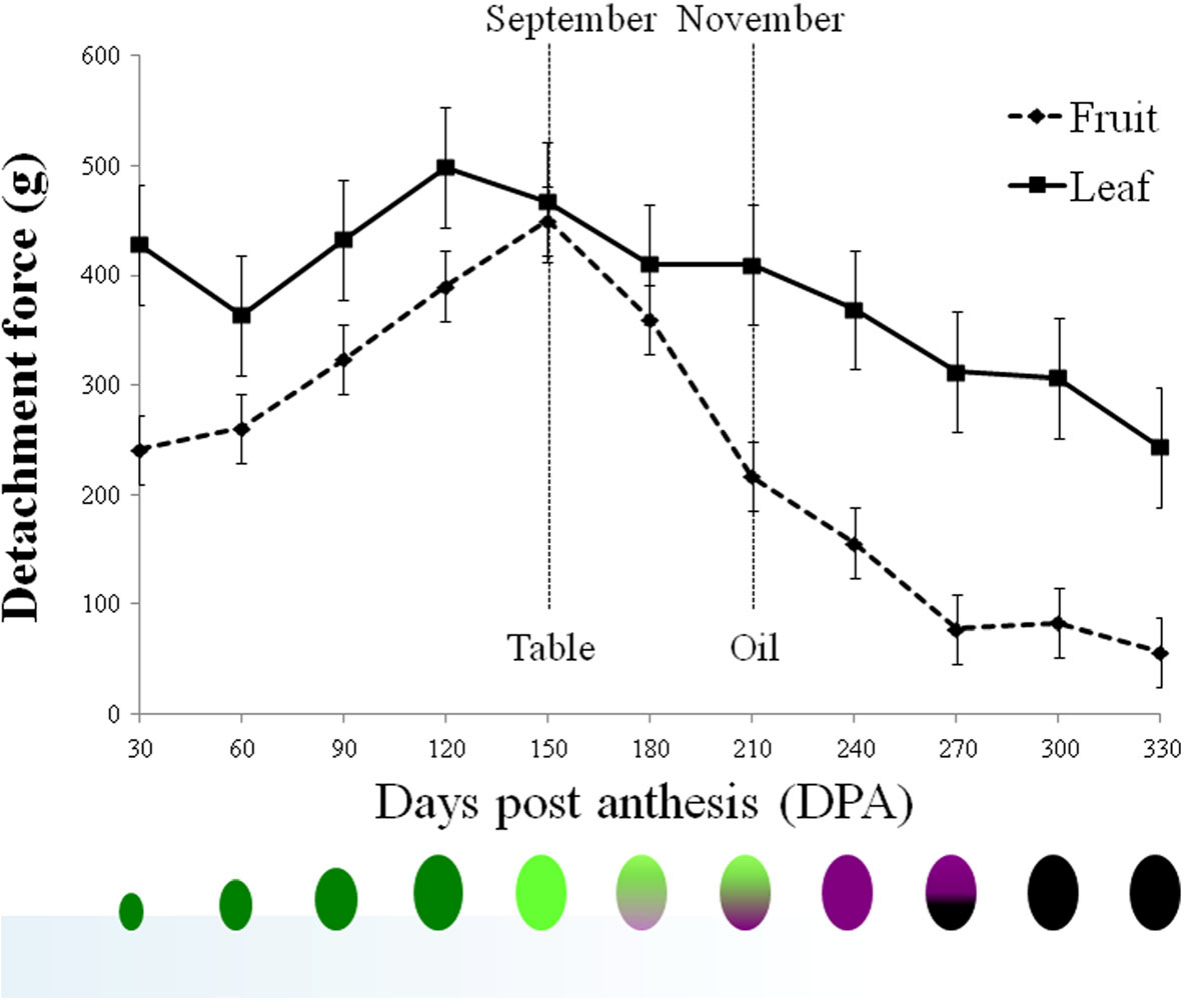
Annual kinetics of changes in fruit and leaf detachment force (DF) after anthesis. The data are based on monthly DF measurements of 25 leaves and fruits taken from various trees. The samples were taken at the indicated Days Post Anthesis (DPA). The vertical *dashed lines* represent the time of harvest of table and oil olives. *Below* is an illustration of fruit development after anthesis

The annual course of olive (cv. ‘Manzanillo’) leaf and fruit DF was determined from May 2011 till March 2012, based on monthly DF measurements of 25 leaves and fruits taken from various trees (Fig. 1). The DF of olive fruits increased from fruit set in May (30 DPA), until fruits reached their full size in September, the harvest season of table olives. Subsequently, fruit DF began to decrease until it dropped below 100 g in January (270 DPA), when ripening was concluded. The kinetics of the leaf and fruit DFs were similar and only the extent of variation differed. Leaf DF was slightly decreased during fall and winter (180–270 DPA). Leaf and fruit DFs were significantly different during the entire year, except for September and October, which is the harvest season of table olives, during which the fruit and leaf DFs were similar. On the other hand, in November–December (210–240 DPA), which is the oil olive harvest time, the DF of the ripened fruits was significantly lower than that of the leaves.

### Characterization of the three olive AZs

**Fig. 2:**
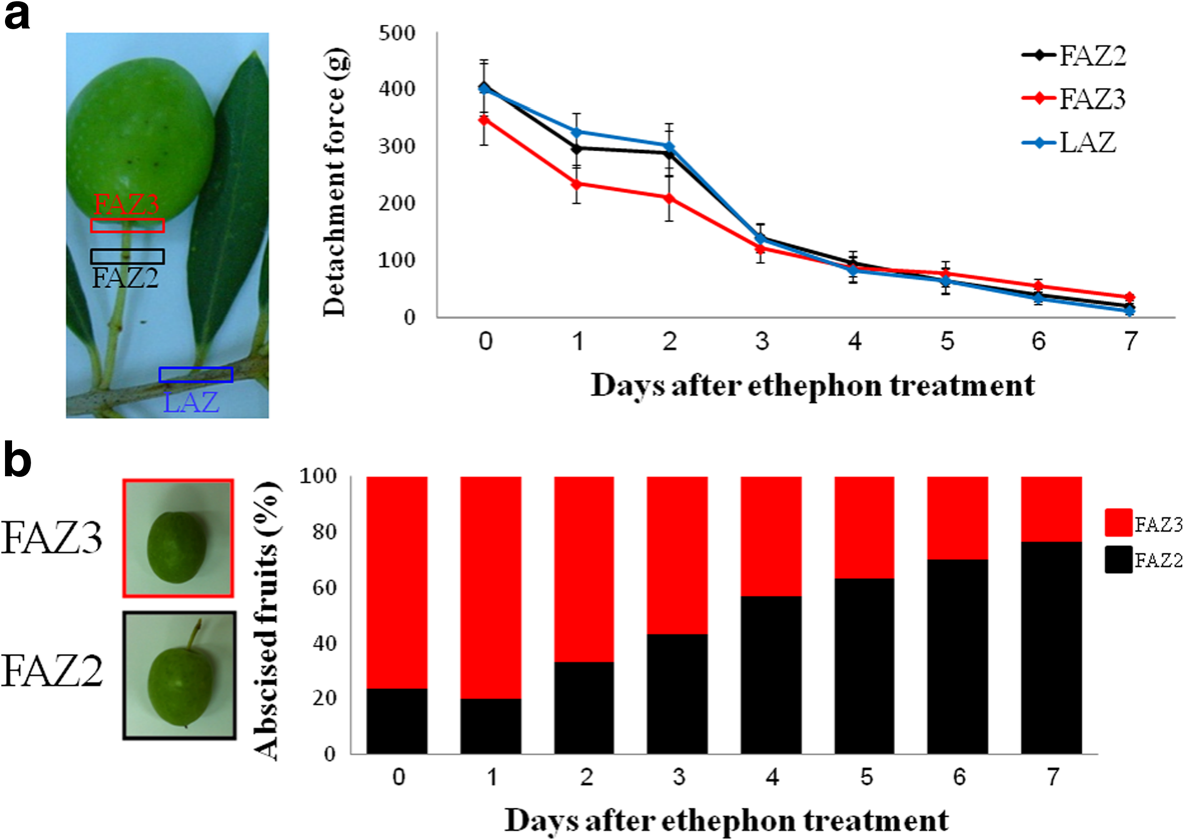
Effect of ethephon treatment on changes in the detachment force of the leaf and fruit AZs (**a**), and on the percentage of abscised fruits from the two fruit AZs (**b**) during 7 days after treatment**.** The location of each AZ is represented by a *rectangle* in the picture on the *left*. Error *bars* in graph (**a**) represent confidence intervals (*p* < 0.95). **b.** The two colored columns represent the percentage of fruits abscised from FAZ2 (*black*) or FAZ3 (*red*) as a function of days after ethephon treatment

In order to examine the effect of ethephon treatment on the DF of fruits and leaves, we treated olive tree branches by applying ethephon at table olive harvest time (September) and conducted daily measurements of the DF of LAZ, FAZ3 and FAZ2 during 7 days after treatment. Since all measurement was done in the field, on detached fruits and leaves and the DF of untreated fruits and leaves was not significantly different during the week after treatment, Day 0 served as a control. On the day of treatment, the DF of LAZ, FAZ2 and FAZ3 were 401, 407 and 350 g, respectively. After treatment, DFs decreased in all AZs continuously; after 4 days DFs had fallen to below 100 g and were at similar levels in all AZs. However, 5 days after treatment, the DF of FAZ2 dropped to 63 compared to 77 g for FAZ3. This trend continued until the end of the experiment, 7 days after treatment, by which time DF of FAZ2 was significantly lower than that of FAZ3 (Fig. 2a). These changes affecting the zone of separation occurred when the fruits were picked: at day 0, 80% of separation took place in FAZ3 and on day 7, about 75% of separation took place in FAZ2 (Fig. 2b).

Our study of the fruit abscission location in cv. ‘Manzanillo’ in September, the table olive harvest time (day 0) showed, that 80% of the fruits abscised at FAZ3 (Fig. 2b), similar to previous findings in olive cv. ‘Hojiblanca’ and ‘Picual’ (Castillo-Lianque and Rapoport, [8]). However, 7 days after ethephon treatment, the fruit abscission position had significantly shifted toward FAZ2 (77%; Prob ChiSq < 0.0006), which was determined to be the major separation zone (Fig. 2b). However, since both FAZs DF had fallen to below 100 g, both can be categorized as ethephon responsive FAZs. We tested the location of fruit abscission in 2-weeks-old fruitlets (14 DPA) and found that the major separation zone at this stage, as in mature fruits, was FAZ3 (68%; Prob ChiSq < 0.01) (data not shown). However, the active AZ during flowering was at FAZ2 (85%; Prob ChiSq < 3.3X10–6). This led us to suggest that FAZ3 is the mature fruit AZ, whereas FAZ2 is the flower-active AZ, which is more responsive to the ethephon treatment in September.

Pectinase activity increased steadily after ethephon treatment in all the three AZ tissues in inverse proportion to the DF. Pectinase activity was greater in the LAZ than in the FAZs at all time points after treatment (Additional file 1: Figure S1). Pectinase activity in the FAZ3 was similar to that of FAZ2, except on day 7 after treatment, where the activity of FAZ2 was significantly higher than that of FAZ3. In water-treated olive branches (control), pectinase activity remained low and stable in all the three AZs (Additional file 1: Figure S1).

### Effect of ethephon treatment on anatomical changes in the olive AZs

**Fig. 3:**
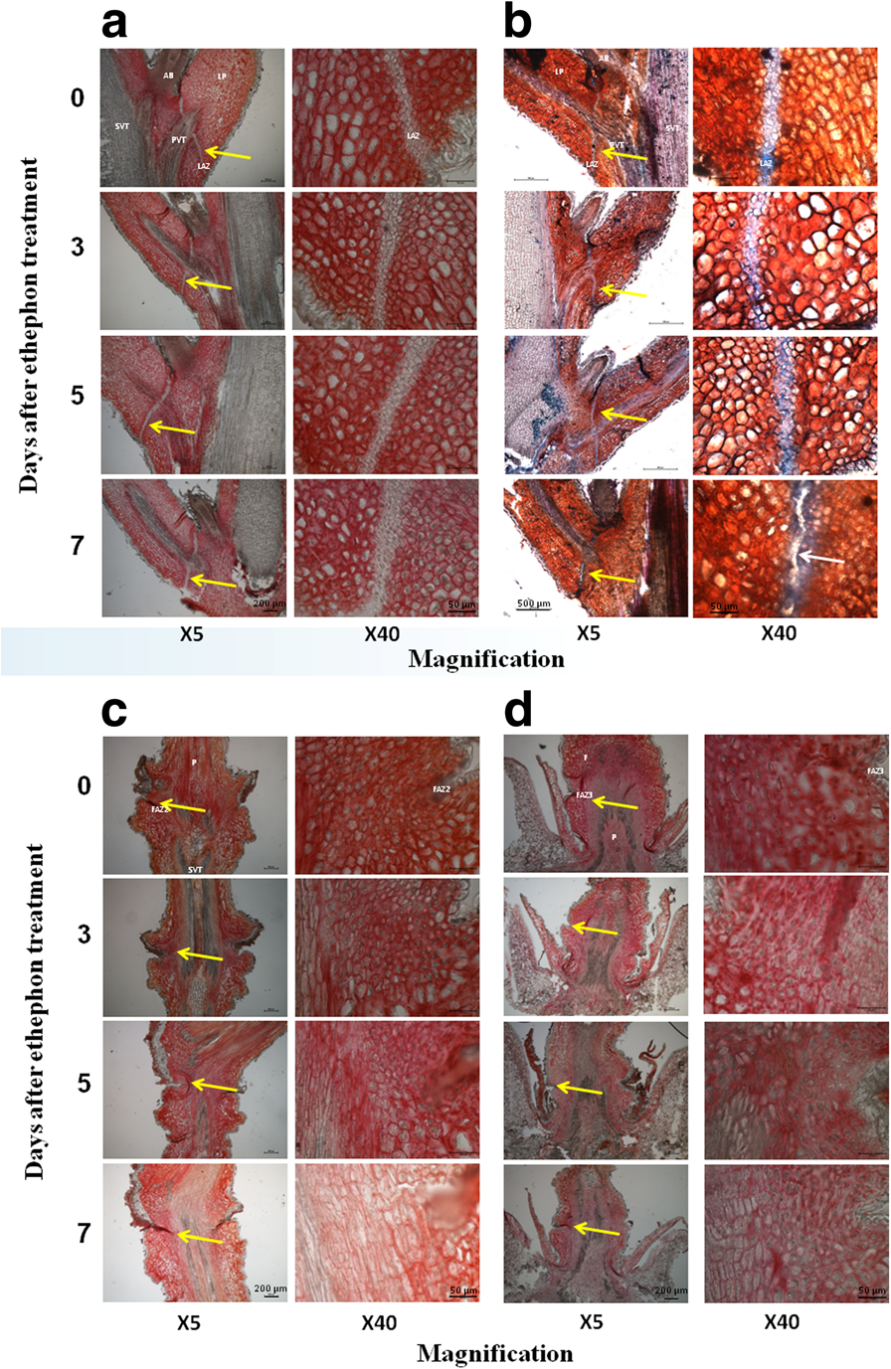
Changes in the anatomy of leaf and fruit AZs during the 7 days after ethephon treatment. Images of longitudinal sections of the LAZ stained with ruthenium *red* (**a**) or alcian *blue* and neutral *red* (**b**), and of fruit FAZ2 (**c**) and FAZ3 (**d**) stained with ruthenium red at ×5 and ×40 magnitudes are presented. Separation of the LAZ 7 days after ethephon treatment is indicated by a *white arrow*. AZs are indicated by *yellow arrows*. The various regions are indicated as follow: LAZ – leaf abscission zone layer, PVT - petiole vascular tissue, AB - axillary bud, LP - leaf petiole, SVT - stem vascular tissue, FAZ2 - fruit abscission zone 2 layer, P – fruit pedicle, FAZ3 - fruit abscission zone 3 layer, F - fruit

Ruthenium red was used to stain the unesterified pectin, in order to identify the abscission zone. The untreated LAZ layer was characterized by layers of small cells with less unesterified pectin compared to neighboring cells (Fig. 3a). Esterified pectin cannot be observed with this stain. The LAZ can be clearly identified in 1-month-old leaves as well as in 2-years-old leaves. It stained very similarly but was characterized by less layers of cells than 1- and 2-years-old leaves (Additional file 2: Figure S2). The 1 month old LAZ mean width is 21.4 μm and significantly (*P* < 1.4X10^−5^) smaller than the width of 1 and 2 years old LAZs (37.4 and 34.7 μm respectively). Cell size was not significantly different (*P* < 0.08) between the LAZs in different ages. FAZ2 and FAZ3 did not contain any specifically defined layer of cells which could be detected by ruthenium red staining. Although we expected degradation of pectin at an early stage of the abscission process, ruthenium red staining did not reveal a decrease in the pectin level in any of the cell layers of the two FAZs, even 7 days after the ethephon treatment. However, tissue separation from the periphery toward the vascular bundle could be detected after ethephon treatment (Fig. 3c and d).

We attempted to monitor cell wall degradation using alcian blue staining, since the dye is excluded by the intact cuticle, and therefore this staining highlights the loss of the cuticle during abscission [46]. Before ethephon treatment (day 0), the alcian blue staining could be detected mainly in the vascular bundle and randomly as small spots over the rest of the cross section. However, distributed staining of AZ tissue was observed 3 days after ethephon treatment. At this stage, the stain was concentrated in the outer layer of the AZ. Five days after treatment, the entire AZ layer was stained by the alcian blue, and 7 days after treatment the alcian blue staining was at its peak, and tissue separation could be clearly seen (Fig. 3b).

### Effect of ethephon treatment on transcriptomic changes in olive AZs

**Fig. 4:**
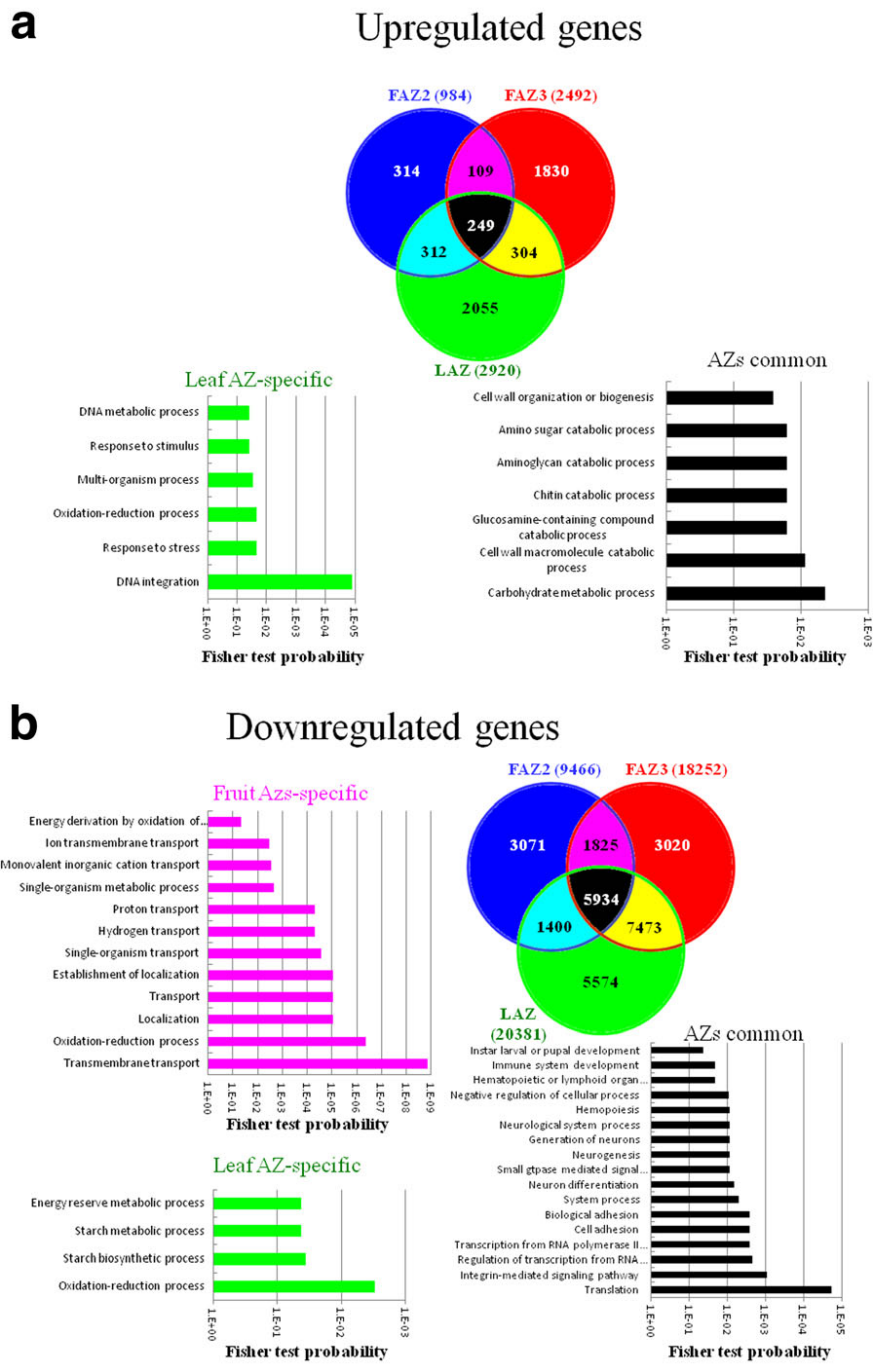
Differentially expressed contigs in the three different AZs 7 days after ethephon treatment. Venn diagrams of upregulated (**a**) and downregulated (**b**) contigs in response to ethephon treatment are presented. The number of contigs significantly regulated in each of the AZs is indicated inside the *circles*. Significant GO enriched biological processes are presented for the common contigs regulated in all three AZs (*black*), for contigs specifically regulated in the LAZ (*green*), and for contigs specifically regulated in the FAZ2 and FAZ3 (*pink*). Among the 109 common contigs upregulated in the FAZ2 and FAZ3, none of the GOs was significantly enriched

In order to identify different molecular signals which distinguish the leaf and the fruit abscission process in response to ethephon treatment, we analyzed the transcriptome profiles of the three olive AZs. RNA was extracted from FAZ3, FAZ2 and LAZ before ethephon treatment and 5 days after, then sequenced using Illumina technology. We obtained an average of 34,347,262 raw reads from each of the six analyzed AZs with 80.5% mapping (Additional file 3: Table S1). The sequencing data were deposited in the NCBI Sequence Read Archive (SRA) database as bioproject PRJNA342541 (SRR accessions: SRR4236909, SRR4236910, SRR4236911, SRR4236912, SRR4236913 and SRR4236914 for FAZ2–0, FAZ2–5, FAZ3–0, FAZ3–5, LAZ-0 and LAZ-5, respectively; https://www.ncbi.nlm.nih.gov/sra?linkname=bioproject_sra_all&from_uid=342541). These sequences were assembled into a total of 138,824 transcripts with a contig N50 of 894 bp and an average contig length of 593 bp. Transcripts UniProt identities were then found by performing a BLAST analysis of the assembled sequences. Hierarchical clustering of all transcripts revealed that gene expression profiles of the two FAZs - FAZ2 and FAZ3, were closer to each other than to the LAZ. In addition, samples were separated into two cluster groups: control AZs and all ethephon-treated AZs. Most of the differentially expressed transcripts were downregulated 5 days after ethephon treatment (Additional file 4: Figure S3). Specifically, among all 138,824 transcripts, 34,100 were differentially expressed in response to ethephon application. Of these, most of the regulated transcripts were downregulated (28,927 transcripts). Only 5173 transcripts were upregulated in response to ethephon treatment in all three AZs (Fig. 4a). Among the differentially expressed genes, more genes were up- or down-regulated in the LAZ compared to those in the FAZ3, whereas FAZ2 had the lowest rate of modified genes. Possibly, because 5 days after ethephon treatment, abscission of FAZ2 has started and most of the cells in FAZ2 were already dead. Most of the upregulated genes in response to ethephon were not overlapped (81%) (Fig. 4a), whereas only 40% of the downregulated genes were not overlapped and the different AZs shared 60% of the downregulated genes (Fig. 4b).

In order to analyze the biological functions regulated in the AZs in response to ethephon treatment, we performed a GO enrichment analysis of the various regulated transcript groups. In the upregulated as well as the downregulated transcripts in each of the three AZs, we found enrichment of many GO terms. As expected, the list of upregulated transcripts in all three AZs showed enrichment of transcripts involved in “cell wall macromolecule catabolic process” and “cell wall organization or biogenesis”. None of the GO terms was enriched among the transcripts that were uniquely upregulated in the FAZs. However, among the 2055 transcripts that were upregulated only in the LAZ, GO terms such as “DNA metabolic process”, “response to stimulus”, “response to stress” and “oxidation-reduction process” were found to be enriched (Fig. 4a). Among the transcripts that were uniquely downregulated in the FAZs, we have identified enrichment of transcripts involved in transport processes. SEA resulted in four GO terms enriched among the transcripts that were downregulated in the LAZ alone. These included “energy reserve metabolic process”, “glycogen biosynthetic process”, “glycogen metabolic process” and “oxidation-reduction process”. The downregulated transcripts common to all three AZs were enriched in many GO terms including “biological adhesion” and “cell adhesion” (Fig. 4b).

### Effect of ethephon treatment on the expression patterns of cell wall degrading genes

Leaf and fruit abscission requires the activity of cell wall degrading enzymes.

**Fig. 5:**
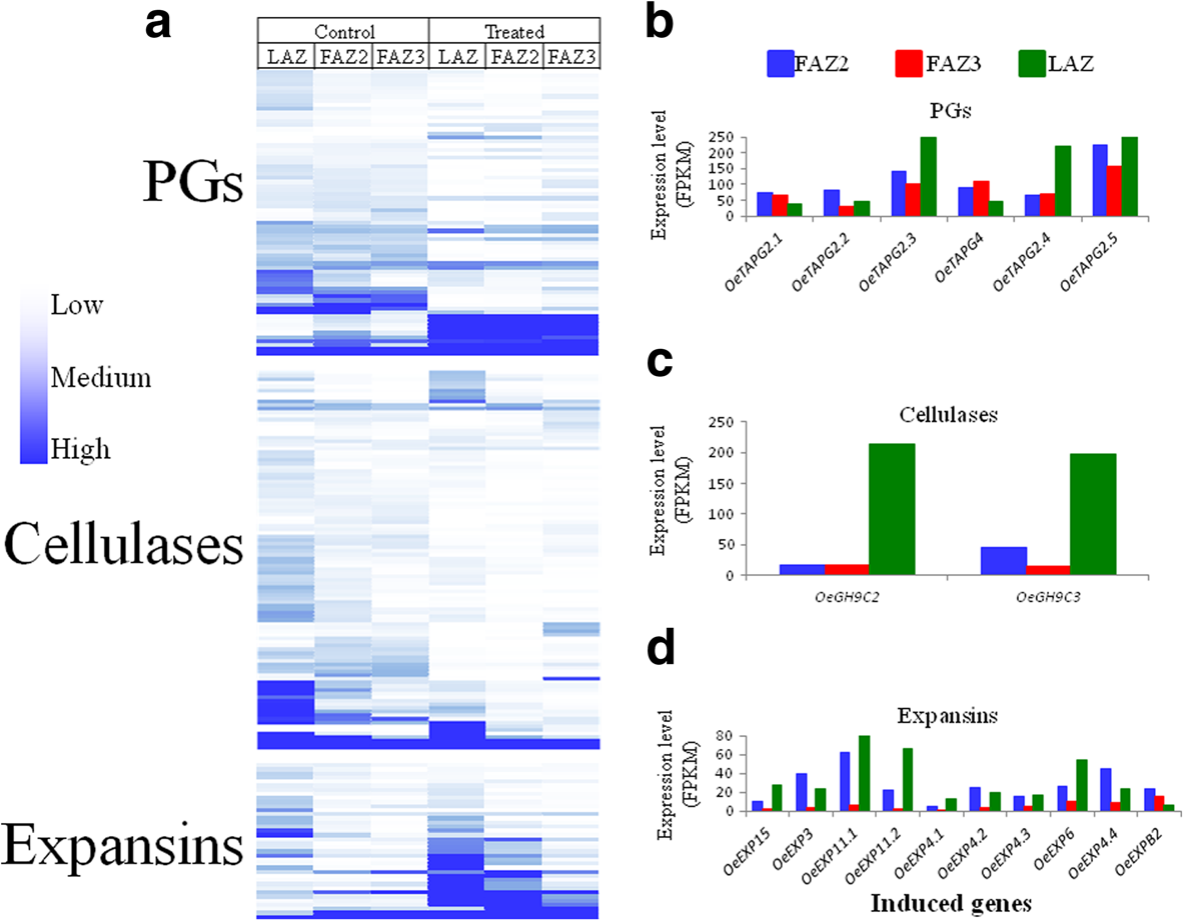
Effect of ethephon treatment on the expression patterns of genes encoding three families of cell wall hydrolyzing enzymes in the three AZs. The expression pattern of all transcripts belonging to PG, cellulase and expansin gene families in the three AZs at 0 and 5 days after ethephon treatment (**a**), and the expression pattern of only the induced genes of PG (**b**), cellulase (**c**) and expansin (**d**) 5 days after the ethephon treatment, are presented

The three main active cell wall degrading enzymes in the plant abscission process are Polygalacturonases (PGs), β-1,4-glucanases (Cellulases or EGs) and Expansins [11]. Therefore, we analyzed the expression patterns of genes belonging to these three families of cell wall degrading enzymes (Fig. 5a). The abundance of most genes of the three families was fairly low (FPKM of 0–2) in all three AZs before and 5 days after ethephon treatment (Fig. 5a). Of these, several genes of each family were induced in response to ethephon treatment (Fig. 5b-d). The six ethephon- induced PG genes showed similar expression patterns in all three AZs (Fig. 5b). Two cellulase genes, *GLYCOSYL HYDROLASE 9C2* (*OeGH9C2*) and *GLYCOSYL HYDROLASE 9C2* (*OeGH9C3*), were dramatically induced by ethephon treatment in the LAZ by approximately 200-fold, and only by about 15-fold in the two FAZs (Fig. 5c). Most of the 10 ethephon-induced expansin genes were highly upregulated in both the LAZ and the FAZ2 compared to their low induction in the FAZ3 (Fig. 5d).

### Effect of ethephon treatment on expression pattern of genes involved in biosynthesis of ethylene, IAA and ABA and in response to these hormones

**Fig. 6:**
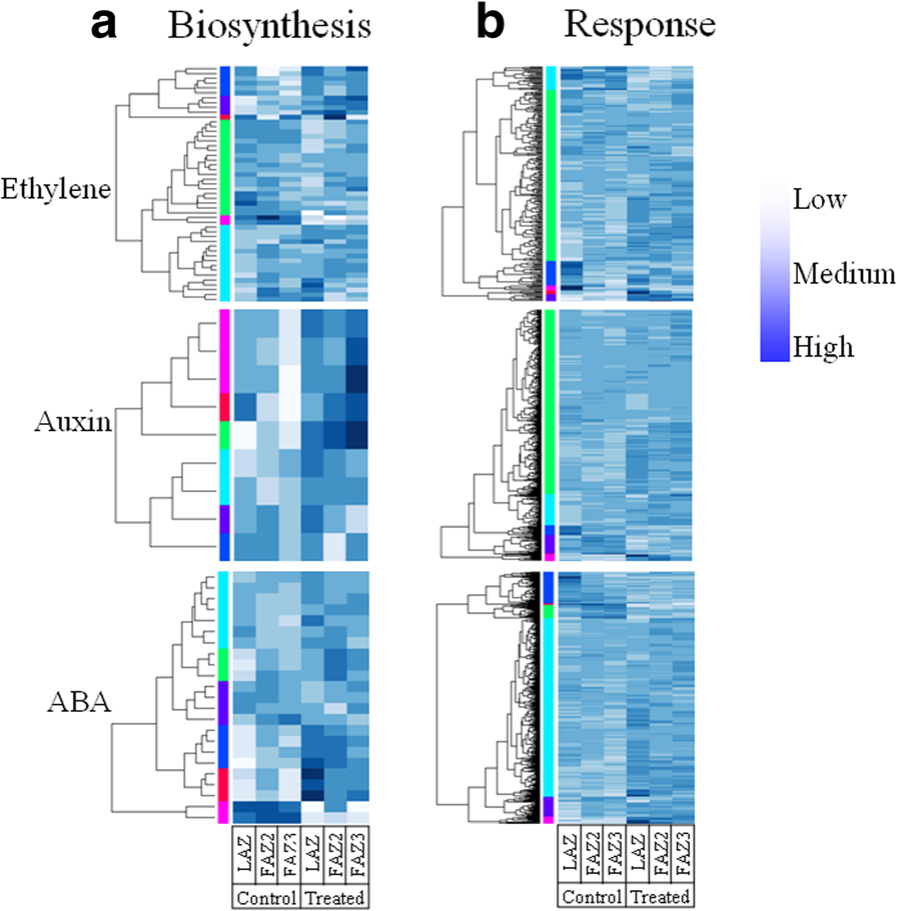
Expression patterns of genes involved in the biosynthesis of and response to the plant hormones ethylene, auxin and ABA in the three AZs before and 5 days after ethephon treatment. Hierarchical clustering of the genes involved in the biosynthesis of (**a**) and the response to (**b**) the hormones in a *green* to *red* scale are presented

The three main plant hormones known to be involved in the abscission process are ethylene, ABA, both accelerating abscission, and auxin, the latter known to delay abscission [47]. Therefore, we analyzed the expression pattern of genes involved in the biosynthesis and the response to these hormones (Additional file 5: Table S2). The results presented in Fig. 6 show the expression of all the transcripts associated with the GO terms for ethylene, auxin and ABA biosynthetic process (GO:000969, GO:0009851, GO:0009688, respectively), as well as to the response to ethylene, auxin and ABA (GO:0009723, GO:0009733, GO:0009737, respectively). Analysis of the abundance of these transcripts in all three AZs 5 days after ethephon treatment, compared to the untreated controls, was carried out. Genes involved in the biosynthesis of auxin and ABA and the response to these hormones were significantly upregulated in response to ethephon treatment. However, only genes involved in the response to ethylene, but not those involved in its biosynthesis, were significantly upregulated (Additional file 6: Table S3). We could not clearly identify tissue-specificity of the genes belonging to the various gene ontology biological processes.

In order to analyze genes involved in the ethylene biosynthesis pathway, we focused on the genes encoding the three main enzymes of the pathway, S-adenosyl methionine synthase (SAMS), 1-aminocyclopropane-1-carboxylate synthase (ACS), and 1-aminocyclopropane-xoxidase (ACO). The expression patterns of *OeSAM*, *OeACS*, and *OeACO* gene families are presented in Additional file 7: Figure S4. Of these, the most abundant genes were found to be *METHIONINE ADENOSYLTRANSFERASES 1* (*OeMAT1*), *OeACS2*, *OeACO1* and *OeACO4*. In the LAZ, the expression of *OeMAT1* and *OeACS2* was downregulated in response to ethephon treatment, whereas the expression of *OeACO1* and *OeACO4* was enhanced. In the FAZs we could not identify significant regulation of any ethylene biosynthesis- related genes other than *OeACS2*, which was downregulated in FAZ2 in response to ethephon treatment (Additional file 7: Figure S4). Among the transcripts associated with the GO term of the auxin biosynthetic process, we found that *ANTHRANILATE SYNTHASE BETA SUBUNIT 1* (*OeASB1*) expression was induced in response to ethephon treatment in all three AZs. Among the transcripts associated with the ABA biosynthetic process, expression of four transcripts (c10724, c34560, c18527, c9486), representing the gene *ZEAXANTHIN EPOXIDASE* (*OeZEP*), were upregulated in all three AZs in response to ethephon treatment. Genes involved in the response to all three hormones included many genes, which most of which were upregulated in all three AZs in response to ethephon treatment. The list of genes involved in the perception and response to ethylene includes *CONSTITUTIVE TRIPLE RESPONSE 1* (*OeCTR1*), *ETHYLENE INSENSITIVE 3* (*OeEIN3*) and many GRAS, NAC and MYB transcription factors known to be ethylene related. Among the transcripts associated with the GO term of the auxin response process are several auxin-responsive *GRETCHEN HAGEN 3* (*GH3*) genes such as *OeGH3.1*, *OeGH3.3* and *OeGH3.6*. (Additional file 8: Figure S5). This list of transcripts also includes the auxin-induced protein *INDOLE-3-ACETIC ACID 13* (*OeIAA13*), *AUXIN RESPONSE FACTORS* (*OeARF5*, *OeARF6*, *OeARF7*) and the auxin biosynthesis gene *ANTHRANILATE SYNTHASE ALPHA SUBUNIT 1* (*OeASA1*). The list of genes involved in the response to ABA includes the transcription factors *ABSCISIC ACID RESPONSIVE ELEMENTS BINDING FACTOR 2* (*OeABF2*) and the ABA-inducible *ABA INDUCIBLE BHLH* (*OeAIB*). It also includes the kinase *CBL INTERACTING PROTEIN KINASE 1* (*OeCIPK1*) and many more (data not shown).

### Differential involvement of reactive oxygen speices (ROS) in leaf and fruit abscission

One of the enriched GO terms in the leaf-specific upregulated transcripts was “oxidation-reduction process” (Fig. 4a). In addition, three out of four of the enriched GO terms among the leaf-specific downregulated transcripts were energy and starch metabolic and biosynthesis processes (Fig. 4b). We also found that application of ethephon induced stomatal closure (data not shown). This raises the possibility that ethephon treatment induces ROS production, which in turn induces oxidative stress and leaf stomatal closure [48].

**Fig. 7:**
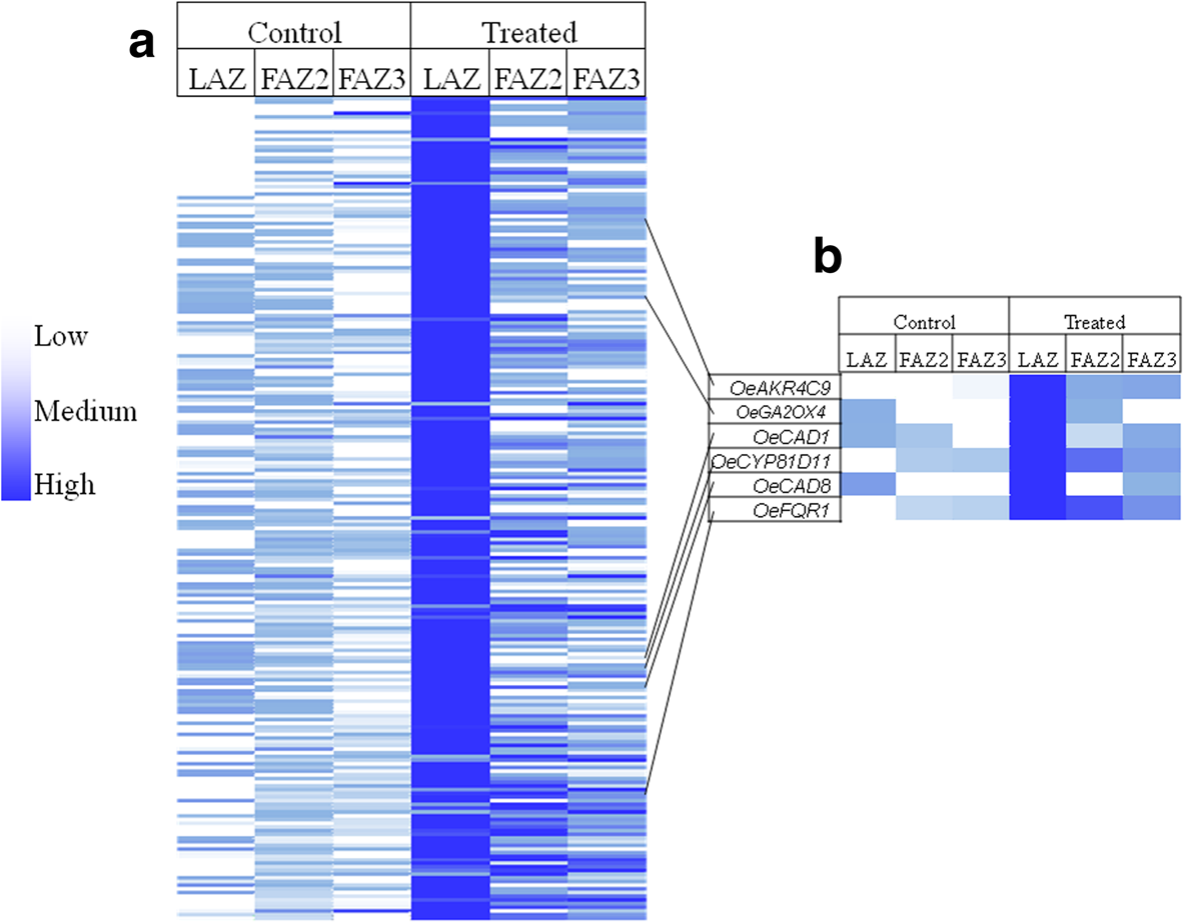
Specific induction in the LAZ by ethephon of genes involved in oxidative stress (**a**) and in response to ROS (**b**). Gene expression patterns of 224 genes involved in oxidative stress (**a**) and of six genes involved in the cell response to ROS (**b**), which were significantly induced in in the LAZ in response to ethephon treatment. Expression levels are indicated on an abundance scale of *green* to *red*. **b**. The expression pattern of the genes *Aldo-Keto Reductase family 4 member C9* (*OeAKR4C9*)*, Gibberellin 2-Oxidase 4* (*OeGA2OX4*)*, Cinnamyl-Alcohol Dehydrogenase 1* (*OeCAD1*)*, Cytochrome P450 81D11* (*OeCYP81D11*)*, Cinnamyl-Alcohol Dehydrogenase 8* (*OeCAD8*) *and Flavodoxin-Like Quinone Reductase 1* (*OeFQR1*), all known to be involved in the cell response to stress is presented

Ethephon treatment specifically and significantly induced the LAZ 224 genes involved in oxidative stress (Fig. 7a). The induced genes included those involved in many aspects of oxidative stress such as: the oxidative phosphorylation pathway –*ATPase F-TYPE SUBUNIT B* (*OeATPF0B*) and *NADH DEHYDROGENASE SUBUNIT 4 L* (*OeND4L*); peroxisome biogenesis - the fatty acid oxidation genes *ACYL-CoA OXIDASE* (*OeACOX*) and *PEROXISOMAL 2,4-DIENOYL CoA REDUCTASES* (*OePDCR*) and the amino acid metabolism gene *ISOCITRATE DEHYDROGENASE* (*OeIDH*); Phaseic acid biosynthesis genes *CYTOCHROME P450* (*OeCYP707A3* and *OeCYP707A1*); Superoxide radicals degradation genes *CATALASE* (*OeCAT2*, *OeCAT3*) and *SUPEROXIDE DISMUTASE 1* (*OeSOD1*) and genes involved in the pentose phosphate pathway – *GLUCOSE 6 PHOSPHATE DEHYDROGENASE* (*OeG6PD2* and *OeG6PD5*). ROS can be generated by various enzymatic activities, and blocked by an array of ROS-scavenging enzymes [49]. The expression of *AMINE OXIDASE 1* (*OeAO1*) was significantly upregulated in the LAZ but not in the two FAZs. Among the 224 genes involved in oxidative stress, several genes are known to be induced by ROS. Those genes consist of: *GIBBERELLIN 2 OXIDASE 4* (*OeGA2OX4*), *CINNAMYL ALCOHOL DEHYDROGENASE 1* (*OeCAD1*), *CINNAMYL ALCOHOL DEHYDROGENASE 1* (*OeCAD8*), *ALDO-KETO REDUCTASE 4-C9* (*OeAKR4C9*), *CYTOCHROME P450* (*OeCYP81D11*) and *FLAVODOXIN LIKE QUINONE REDUCTASE 1* (*OeFQR1*) (Fig. 7b).

**Fig. 8:**
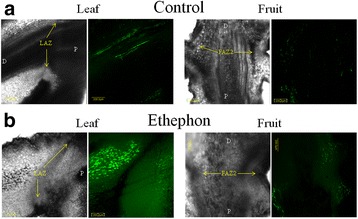
Effect of ethephon treatment on ROS formation in the leaf and fruit AZs before (**a**) and 3 days after (**b**) treatment. DCF fluorescence under a confocal scanning microscope of the LAZ and FAZ2 is presented. The location of the AZs is indicated by *yellow arrows*. P, proximal side; D, distal side

**Fig. 9:**
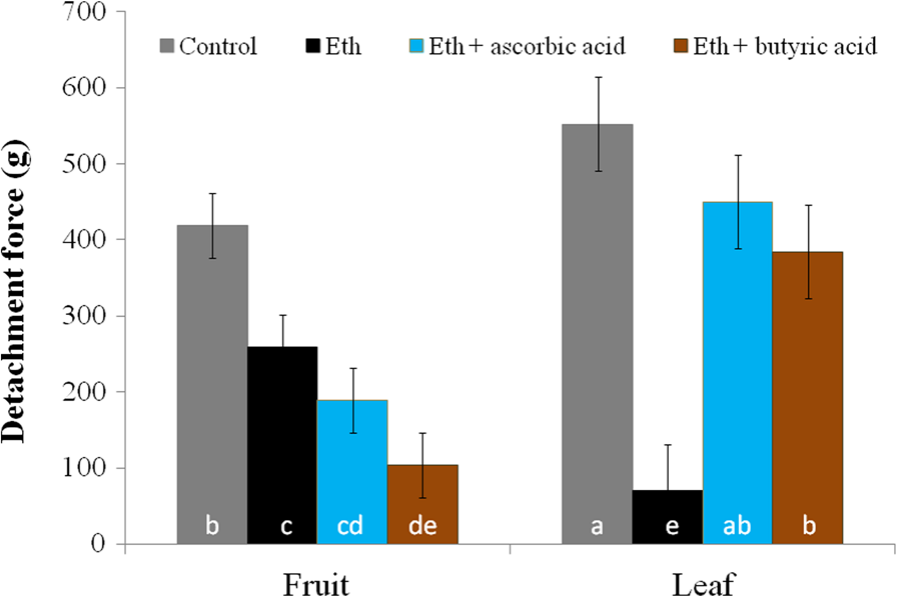
Effect of a combined treatment of ethephon and antioxidants on the DF of leaves and fruits 3 days after treatment. Error bars represent confidence intervals based on 30 samples (*p* < 0.95) and different letters represent statistically different DF

We used DCF as a ROS indicator to evaluate ROS production in the LAZ and FAZ2 following treatment with ethephon. Pedicels of the untreated leaf and fruit showed a very weak DCF fluorescence. For the first 2 days after treatment, there was no significant rise in ROS production in either of the AZs (data not shown). However, on the third day after ethephon treatment, DCF fluorescence had increased 4-fold in the leaf pedicel but not in the FAZ2 (fluorescence intensities of 54 vs. 12 respectively; *P* < 0.0005) (Fig. 8b). The DCF fluorescence in the leaf after ethephon treatment showed higher intensity in the distal compared to the proximal side, whereas no signal could be observed in the AZ cells (Fig. 8b). Since ROS production in response to ethephon was observed in LAZ and not in the FAZs, we speculated that ethephon induced abscission of leaves might be ROS dependent. Therefore, we treated olive leaves and fruits with antioxidant added to the ethephon and compared their response. We found that application of ethephon mixed with an antioxidant repressed the ethephon-generated effect of stomatal closure as well as reducing the ROS fluorescence intensity to the value of an untreated leaf (data not shown). Accordingly, the addition of antioxidants such as 0.3% ascorbic acid or 100 mM butyric acid inhibited the reduction of the DF in response to ethephon in leaves but not in fruits. The leaf DF was 552 g and decreased down to 70 g after ethephon treatment. However, in a response to ethephon mixed with butyric acid, leaf DF decreased only to 384 g, whereas in a response to ethephon mixed with ascorbic acid, leaf DF was 450 g and was not significantly different from the control leaf DF. Conversely, adding antioxidants to ethephon reduced the fruit DF and thereby enhanced fruit abscission. The fruit DF was 419 g and decreased to 259 g after ethephon treatment. In a response to ethephon mixed with ascorbic acid, fruit DF was 189 g and was not significantly different from the control fruit DF. However, in a response to ethephon mixed with butyric acid fruit DF decreased to 104 g, which was found to be significantly lower than fruit DF in a response to ethephon (Fig. 9).

## Discussion

Significant research has been devoted to adapt mechanical harvesting for table olives [31]. A detailed characterization of the molecular differences between the olive FAZs and LAZ should facilitate the development of a selective abscission treatment, reducing DF of fruit but not of leaves, thus enabling mechanical harvesting of table olives.

### Differential activity of the olive FAZs during fruit development

The olive (*Olea europaea* L.) fruit has two FAZs. Our study suggests that the AZ between the pedicel and the rachis (FAZ2) is active only during flowering. Upon fruit set, activity shifts to the mature fruit AZ between the pedicel and fruit (FAZ3). The immature fruit AZ (FAZ2) is highly sensitive to exogenous ethylene, whereas the mature fruit AZ (FAZ3) is also sensitive to exogenous ethylene but to a lesser degree than FAZ2 (Fig. 2). Similarly, langsat (*Lansium domesticum*) fruit has two AZs: one located between the stem and the fused sepals (FAZ2), and the other between the fused sepals and the fruit (FAZ3). FAZ3 is the active AZ during the ripe fruit stage, whereas FAZ2 is active in young as well as in mature fruit. Unlike in olives, these two AZs differ in their sensitivity to exogenous ethylene as well. FAZ2 is highly sensitive, whereas FAZ3 in langsat fruit is less sensitive to ethylene treatment [5]. Citrus fruits also have two AZs that differ in their activity during fruit development: AZ-A is active at an early stage of fruit development and AZ-C at a later stage [50]. Peaches have three AZs, one active in the early stage of fruit development, and the other two in the late stage [51]. Reed and Hartmann [52] found that following the application of 2-chloroethyl-tris-(2-methoxyethoxy)-silane (CGA 13586), an ethylene-releasing compound, many morphological changes occurred in the olive fruit abscission zone. They observed changes in the cell plasmolysis and cell wall, middle lamella dissolution, starch grain accumulation and general breakdown of cells at the fruit abscission zone. We could not identify these morphological changes after ethephon treatment.

### Ethephon-induced fruit abscission differs from natural abscission

Gil-Amado and Gomez-Jimenez [29] compared the olive cv. ‘Picual’ FAZ3 transcriptomes present at the pre- abscission stage (154 DPA) to those of the mature abscission stage (217 DPA). They found that membrane microdomains involving sphingolipids and remorins, together with signaling proteins, are involved in the abscission process. Their findings also suggest the involvement of long-chain base metabolism and small GTPases in regulating cell wall modifications and abscission. Although their study characterized natural abscission processes and ours characterized an ethylene-induced abscission process in olives, both studies compared the transcriptomes of inactive FAZs to those of active ones. Both studies also used tissues enriched with AZs without any non-AZ control. Gil-Amado and Gomez-Jimenez clustered the differentially expressed genes into four clusters according to their abundance: A1 (repressed at 217 DPA with some expression at 217 DPA), A2 (repressed at 217 DPA with no expression), B1 (induced at 217 DPA with some expression at 154 DPA) and B2 (induced at 217 DPA with no expression at 154 DPA). We compared the expression pattern of the top 10 regulated genes in each of the four clusters to the expression patterns in the three AZs (LAZ, FAZ2 and FAZ3) before, and 5 days after ethephon treatment. We found that in most cases, the genes regulated during natural abscission did not respond to ethephon. These genes including *RAN GTPase 3*, *ENDOMEMBRANE-TYPE Ca-ATPase 4*, *PURPLE ACID PHOSPHATASE 18* (*OePAP18*), *PROTEIN PHOSPHATASE 2C*, *ACC OXIDASE*, *ATP CITRATE LYASE*, *PHENYLALANINE AMMONIA-LYASE*, *CYTOCHROME P450* and others, are all upregulated during ripening but did not respond to ethephon in all tested AZs. In addition, genes such as *CALMODULIN-7* (*OeCAM7*), *OXYGEN-EVOLVING ENHANCER PROTEIN 1* (*OeOEE1*), *CYTOCHROME B6-F COMPLEX SUBUNIT 4* (*OePETD*), *SERINE-THREONINE PROTEIN KINASE*, *CHLOROPLAST HSP70* and others, are all downregulated during ripening but did not responded to ethephon in all tested AZs (Additional file 8: Figure S5). The correlation between the transcription levels in FAZ3 in response to ethephon was low (*r* = 0.254) and not statistically significant (*P* < 0.109). We would like to suggest that although the abscission process is similar, FAZ activation in the olive fruit abscission process by means of exogenous ethylene treatment differs from the activation of the natural abscission process, which occurs in response to ripening. It is also possible that other dosage of ethephon would have results in a similar activation as the natural abscission process, since it was already shown that ethephon dosage affect the abscission process [53].

### Differential induction by ethephon of genes encoding cell wall degrading enzymes in the three AZs

As expected, our GO enrichment test revealed upregulation of genes involved in cell wall organization and cell wall macromolecule catabolic processes in all three AZs in response to ethephon. The main cell wall degrading enzymes active during abscission are cellulase, PG, and expansin [19, 54–57]. Our results suggest that although genes encoding cell wall degrading enzymes are activated in all three analyzed olive AZs, their expression differ in the various AZs. PG genes are expressed in all the three analyzed AZ tissues (Fig. 5b). On the other hand, the expression of cellulase genes is strongly LAZ-specific (Fig. 5c), while the expression of expansin genes are LAZ- and FAZ2-specific (Fig. 5d), and are minimally expressed in the FAZ3 (Fig. 5c, d). We found that olive FAZ2 is more sensitive to ethephon compared to FAZ3. Indeed, the expression level of most of the genes encoding cell wall degrading enzymes, including *OeTAG2.1*, *OeTAG2.2*, *OeTAG2.3*, *OeTAG2.5*, *OeGH9C3* and all 10 expansins, is higher in FAZ2 compared to their expression in FAZ3 (Fig. 5). PGs were shown to be induced in response to exogenous ethylene in tomato leaf and flower AZs [58]. Another study [24], comparing the global transcriptomes generated from the FAZ and the LAZ of tomato plants during abscission revealed that PGs were induced in both AZs in response to organ removal. However, in line with our results in olives, the expression of most cellulase and expansin genes was higher in the LAZ compared to their expression in the FAZ.

### Ethephon does not affect ethylene biosynthesis but induces ethylene signal transduction

We could not detect any endogenous ethylene production in olive leaves or fruits following ethephon treatment (data not shown). Measurements of endogenous ethylene production in olive leaves and fruits once a month, all year round, including in fruits left on the tree a long time after ripening, did not identify ethylene production in leaves or in fruits (data not shown). In addition, genes involved in ethylene biosynthesis were not induced following ethephon treatment (Fig. 6a). However, genes involved in response to ethylene were induced in response to ethephon treatment (Fig. 6b). Unlike this situation in olives, ethylene biosynthesis increases before abscission in many shedding organs, including reproductive organs [59]. In woody plants, a role for ethylene in abscission has largely been confirmed by application of exogenous ethylene (ethephon) and its precursors (Sawicki et al., [60]). Autocatalysis of ethylene production is a characteristic feature of ripening fruits and other senescing tissues, in which a massive increase in endogenous ethylene production is triggered by exposure to ethylene [61]. In apple fruitlets, the induction of abscission using thinning chemicals stimulated ethylene biosynthesis in parallel with the upregulation of key regulatory genes [62–64]. This regulation was also reported in grapevines during flower and fruit abscission [65]. Autocatalytic ethylene production was found in many plants including tomato [66], *Dendrobium* flowers [67], citrus [68], Kiwifruit [69] and others. We found that the only genes upregulated in response to ethephon are ACC oxidases involved in the last step of ethylene biosynthesis of ethylene – conversion of ACC to ethylene. This result is in agreement with a previously report that application of 1-aminocyclopropane-1-carboxylic acid (ACC) induced ethylene production and abscission of olive fruits [70]. However, unlike in other fruit tree species, our results may suggest that the ethephon-induced abscission was mediated by release of exogenous ethylene and did not result from induction of endogenous ethylene biosynthesis in response to the ethephon treatment. It is possible that the time of treatment is such that cells/tissues are not able to synthesize ethylene due to their particular stage of development.

### Ethephon induces genes involved in hormonal biosynthesis and activity

Hormonal changes during abscission have been well documented in many studies. Ethylene and ABA act as the major abscission-accelerating signals, while auxin and polyamines are considered as abscission inhibitors [10, 60, 62, 71]. Gibberellin effect on abscission is still unclear. Some reports suggested an abscission inhibiting role [72, 73], while others suggest that gibberellin promotes abscission [74–76]. A large number of genes regulating abscission are also part of the hormone biosynthetic and signaling pathways or influence their metabolism. Auxin has been shown to delay the activation of the AZ by reducing its sensitivity to ethylene [12, 23, 77, 78]. However, auxin application induces ethylene biosynthesis and signaling, leading to fruitlet abscission [79].

The enzyme anthranilate synthase catalyzes the first reaction in the tryptophan biosynthetic pathway [80]**.** Expression level of the two genes *WEAK ETHYLENE INSENSITIVE2* (*WEI2*/*ASA1*) and *WEI7* (*ASB1*), which encode α- and β-subunits of anthranilate synthase, were upregulated in response to ethylene stimulation in Arabidopsis roots [81], leading to increased levels of auxin in the root tips. We found that *OeASB1* expression and other genes involved in auxin biosynthesis, according to their GO characteristics, were induced in response to ethephon treatment in all AZ’s (Fig. 6). Our results suggest crosstalk between ethylene and auxin, in which exogenous ethylene may induce endogenous auxin biosynthesis. However, this endogenous auxin could not prevent abscission as the levels of IAA were not necessarily increased. Among our 20 transcripts encoding genes belonging to the *GH3* gene family, the most abundant one (more than 10-fold more than all other genes in this family) encodes *OeGH3.1*. *OeGH3.1.2* was found to be induced 5-fold in response to ethephon (Additional file 9: Figure S6). It is well established that GH3 enzymes produce IAA conjugates with several amino acids which aid in maintaining auxin homeostasis, both by inactivating IAA and by serving as a reservoir of IAA that can be released upon hydrolysis [82, 83]. Keeping this in mind, our results may suggest that ethylene released by the ethephon treatment could contribute to a certain increment of IAA conjugation, thus affecting auxin homeostasis. However, IAA and conjugated IAA quantification need to be carried out.

The first enzyme to be identified as an ABA biosynthetic enzyme was zeaxanthin epoxidase (ZEP/ABA1), which converts zeaxanthin to violaxanthin [84], a key reaction in ABA biosynthesis [85]. Several ABA biosynthesis genes including *OeZEP*, were upregulated in all the three olive AZs in response to ethephon treatment (Fig. 6a). Increased ABA biosynthesis in response to exogenous ethylene could be expected since crosstalk between these hormones is well documented [86], and ABA is known to accelerate abscission [60]. The transcriptome results suggest that upon exogenous ethylene treatment, all three hormones, ethylene, auxin and ABA act together to induce abscission of the olive fruits and leaves. It was already shown that the induction of the apple fruitlet abscission process at the cortex level is orchestrated by a multiple network of interactions between hormones (mainly ABA and ethylene) and other signaling molecules such as ROS [16]. In our study, although gene expression pattern suggests induction of ABA and auxin in a response to ethephon treatment, hormonal profiling and quantification must be carried out in order to confirm this hypothesis.

### Ethephon induces ROS in the LAZ but not in the FAZ

ROS are a second class of small, versatile molecules that are active in a wide range of cellular processes, including programmed cell death and hormonal signaling. Previous reports have linked ethylene with ROS signaling events [25, 87, 88]. We found that significantly more ROS-related genes were induced in response to ethephon at the LAZ, than in the two FAZs. The main gene family involved in ROS production is the NADPH oxidase gene family, which was not found among our transcripts. However, the expression of *OeAO1*, which produces ROS in apoplastic tissue [89, 90], was significantly upregulated in the LAZ but not in the two FAZs. The ROS signaling network controls a broad range of biological processes, including biotic and abiotic stress responses, by activating defense genes [91]. Among the 224 genes involved in oxidative stress, several genes are known to be induced by ROS and are part of the defense reaction of the plant [92]. Those genes consist of: *OeGA2OX4* which induce gibberellin (GA) turnover and is assumed to have a role in integration of extrinsic signals into the developmental program [93], two genes involved in biosynthesis of lignin (*OeCAD1* and *OeCAD8*) [94], the aldo-keto reductase, *OeAKR4C9*, known to be involved in diverse plant metabolic processes and stress defense [95], the stress defense gene, cytochrome P450, *OeCYP81D11* and the gene involved in flavonoid biosynthesis, *OeFQR1*, known to be induced by ROS [92] (Fig. 7). The ethephon-induced ROS favored the distal rather than the proximal side of the LAZ (Fig. 8b). Plants possess very efficient enzymatic and non-enzymatic antioxidant defense systems, which protect plant cells from oxidative damage by scavenging of ROS [96]. Many antioxidants were shown to effectively repress ROS-induced damage [97–99]. Among them, ascorbic acid [100] and butyric acid [101] were shown to effectively repress ROS damage in leaves. Adding antioxidants to the ethephon treatment inhibited the decrease in the DF of olive leaves, while enhancing the decrease in DF of fruits (Fig. 9). Hartmann et al. [102] also found that spraying ascorbic or iodoacetic acid before harvest causes a reduction in olive fruit detachment force. Therefore, we suggest that olive leaf abscission induced by exogenous ethylene is mediated by ROS, while the pathway responsible for fruit abscission is probably not mediated by ROS. Chilling-induced leaf abscission of *Ixora coccinea* plants was reported to be mediated via oxidative stress, which increased LAZ sensitivity to ethylene. Accordingly, antioxidants were shown to decrease the sensitivity of LAZ to ethylene and thus inhibited leaf abscission, which was correlated to higher IAA levels [103–105]. The effects of ethylene and ROS on IAA depletion and sensitivity of AZs to ethylene has been extensively discussed in a recent review [12]. Agusti et al. [25] suggested that ROS expressed in the citrus LAZ is part of a general defensive program including generation of physical barriers. ROS was also shown to play an important regulatory role in the process of Cassava leaf abscission under water deficit stress [106]. Another study showed that continuous production of hydrogen peroxide (H_2_O_2_) resulted in pepper leaf abscission [14, 107]. However, several studies have suggested a role for ROS in fruit abscission. Carbohydrate stress obtained by girdling induced fruit abscission of longan (*Dimocarpus longan*) mediated by ROS [108]. This study found that H_2_O_2_ was located exclusively in the cell walls of the AZ, 2 days after treatment. It virtually disappeared by the third day after treatment, and reappeared in the mitochondria and cell walls 1 day later. Transcripts belonging to the ROS response were preferentially regulated in the ethephon-treated FAZ of lychee [109]. It was also shown that induced abscission in tomato flowers resulted in a significantly higher ROS level on the distal AZ fracture plane compared with the proximal AZ surface [110].

## Conclusion

Because both fruit and leaf DF levels are high during the olive harvest season, a fruit-specific agent is required to enable mechanical harvesting of table olives. Our study examined various aspects of the distinctive mechanisms characterizing fruit and leaf AZs of table olives. We found that unlike the FAZ, the LAZ is morphologically characterized by small cells containing less pectin compared to neighboring cells.

In this study we have not used the non-AZ region as a control. As a control, we used branches dipped in water instead of the solution of 4% MPK and 0.3% paraffin oil used in the ethephon treatment. However, our main goal was to compare three types of AZs, and their response to the ethephon treatment, in order to define the ethephon-induced changes in gene expression in these AZ tissues. Thus, ethephon may also induce changes in the different NAZ’s of these tissues, but this is irrelevant to the scope of the work. Exogenous ethylene treatment affected the expression of many hormone-related genes in the three AZs. We found that genes involved in biosynthesis of auxin and ABA and in the response to them, were upregulated in response to ethephon treatment in all three analyzed abscission zones. However, only genes involved in the response to ethylene but not in its biosynthesis were upregulated in response to ethephon. In both the LAZ and the FAZs ethephon treatment induced genes encoding cell wall degrading enzymes. However, induction of cellulase genes was specific to the LAZ. Ethephon treatment enhanced pectinase activity and reduced DF in all the three AZs of olives. However, ROS-mediated abscission in response to ethephon is specific to the leaf abscission process, and does not operate in that of fruit. Therefore, addition of an antioxidant such as ascorbic acid or butyric acid to the applied ethephon solution inhibited leaf abscission but enhanced fruit abscission. Future studies should validate that adding the antioxidant does not affect fruit firmness and texture nor does it have phytotoxic effects on leaves remaining on the trees which can affect crop yields. Our findings point the way for developing a commercial application of a differential treatment generating a reduced DF specifically at the FAZ. This would be a significant step in facilitating the mechanical harvesting of table olives.

## Additional files

Additional file 1: Figure S1. Changes in pectinase activity in the three different AZs during 7 days after ethephon treatment. Pectinase activity in the LAZ (blue line), FAZ2 (black) and FAZ3 (red) in control or ethephon-treated branches is presented. Error bars represent confidence intervals based on 5 samples (*p* < 0.95). (TIFF 250 kb)

Additional file 2: Figure S2. Changes in the anatomy of the leaf AZ observed 1 month, 1 or 2 years after leaf appearance. Images of longitudinal sections of the LAZ stained with ruthenium red at ×5, ×10 and ×20 magnitudes are presented. (TIFF 1295 kb)

Additional file 3: Table S1. Deep sequencing and mapping statistics of the three AZs samples before and 5 days after ethephon treatment. (DOCX 14 kb)

Additional file 4: Figure S3. Hierarchical clustering of all transcripts (a) and of differentially expressed transcripts (b) in the three AZs before and 5 days after ethephon treatment. Expression levels are indicated on an abundance scale of green to red. (TIFF 345 kb)

Additional file 5: Table S2. Description and FPKM values of all transcripts involved in biosynthesis of and in response to the plant hormones ethylene, auxin and ABA. (XLSX 151 kb)

Additional file 6: Table S3. Relative abundances of all transcripts involved in biosynthesis of and in response to the plant hormones ethylene, auxin and ABA. Values are the average of logarithmic relative expression (5/0) of all three AZs (FPKM values). A statistical analysis was performed to determine if the average abundances of all transcripts in the three AZs are significantly higher 5 days after treatment as compared to untreated samples. The probability of each test is shown. (DOCX 29 kb)

Additional file 7: Figure S4. Expression of genes involved in the ethylene biosynthesis pathway in the three AZs before and 5 days after ethephon treatment. Expression levels are indicated on an abundance scale of green to red. Only highly abundant transcripts appear with their gene names – *S-Adenosylmethionine Synthase 1* (*OeSAM1; OeMAT1*)*, 1-Amino-Cyclopropane-1-Carboxylate Synthase 2* (*OeACS2*)*, 1-Amino-Cyclopropane-1-Carboxylate Oxidase 4* (*OeACO4*) *and 1-Amino-Cyclopropane-1-Carboxylate Oxidase 1* (*OeACO1*). (TIFF 222 kb)

Additional file 8: Figure S5. Comparison between our results and those of Gil-Amado and Gomez-Jimenez [29]. The expression pattern of the highest ranked genes in each of the four clusters is shown. For each gene (rows) the expression levels in our study appear on the left whereas the expression levels of the same gene in an inactive AZ (154) and active AZ (217) according to Gil-Amado and Gomez-Jimenez appear on the right. (TIFF 172 kb)

Additional file 9: Figure S6. Expression of genes belonging to the *GH3* gene family in the three AZs before and 5 days after ethephon treatment. Expression level is presented in an abundance scale of green to red. The various columns represent the different tissues and treatments as indicated. (TIFF 116 kb)

## Abbreviations

ABA: Abscisic acid
AZ: Abscission zone
DF: Detachment force
DPA: Days post anthesis
FAZ: Fruit abscission zone
GO: Gene ontology
LAZ: Leaf abscission zone
PGs: Polygalacturonases
ROS: Reactive oxygen species
SEA: Singular enrichment analysis
