# Supplementary figures and images for: Ethephon induced oxidative stress in the olive leaf abscission zone enables development of a selective abscission compound
Source: BMC Plant Biol. 2017 May 16;17:87. doi: 10.1186/s12870-017-1035-1 (PMC5434568; doi:10.1186/s12870-017-1035-1)

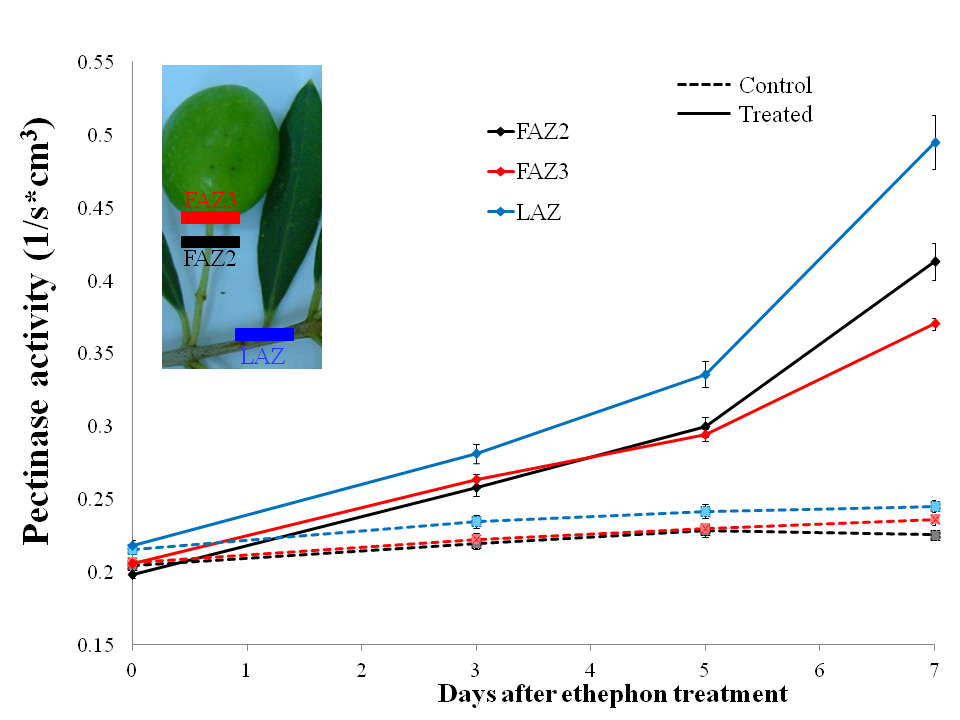

Supplement: Supplementary file 1 — Changes in pectinase activity in the three different AZs during 7 days after ethephon treatment. Pectinase activity in the LAZ (blue line), FAZ2 (black) and FAZ3 (red) in control or ethephon-treated branches is presented. Error bars represent confidence intervals based on 5 samples (p < 0.95). (TIFF 250 kb) [file 12870_2017_1035_MOESM1_ESM.tif]

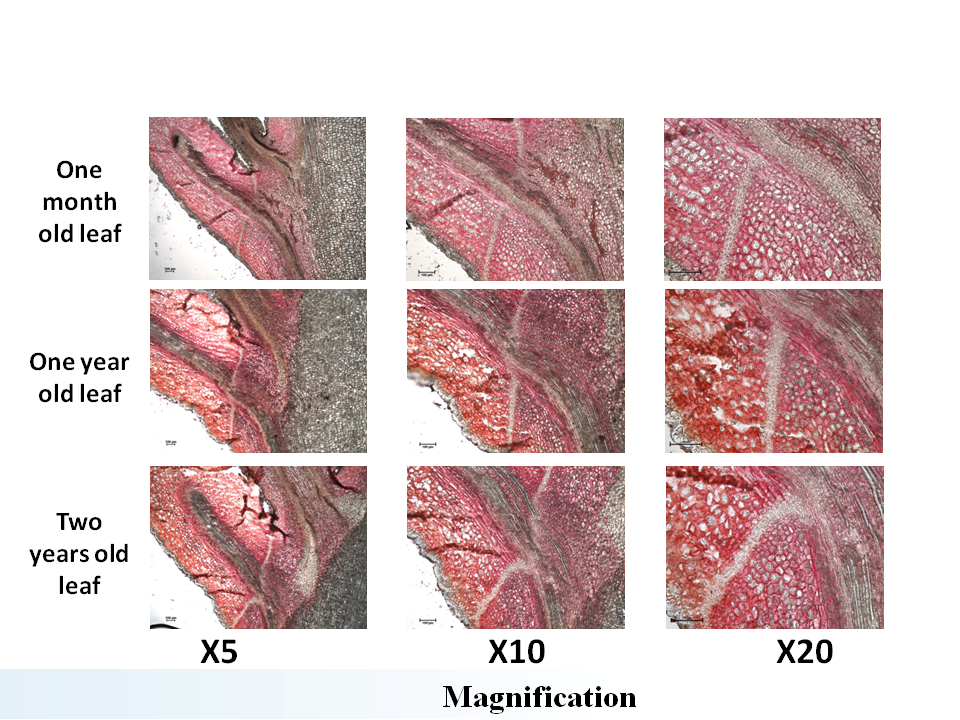

Supplement: Supplementary file 2 — Changes in the anatomy of the leaf AZ observed 1 month, 1 or 2 years after leaf appearance. Images of longitudinal sections of the LAZ stained with ruthenium red at ×5, ×10 and ×20 magnitudes are presented. (TIFF 1295 kb) [file 12870_2017_1035_MOESM2_ESM.tif]

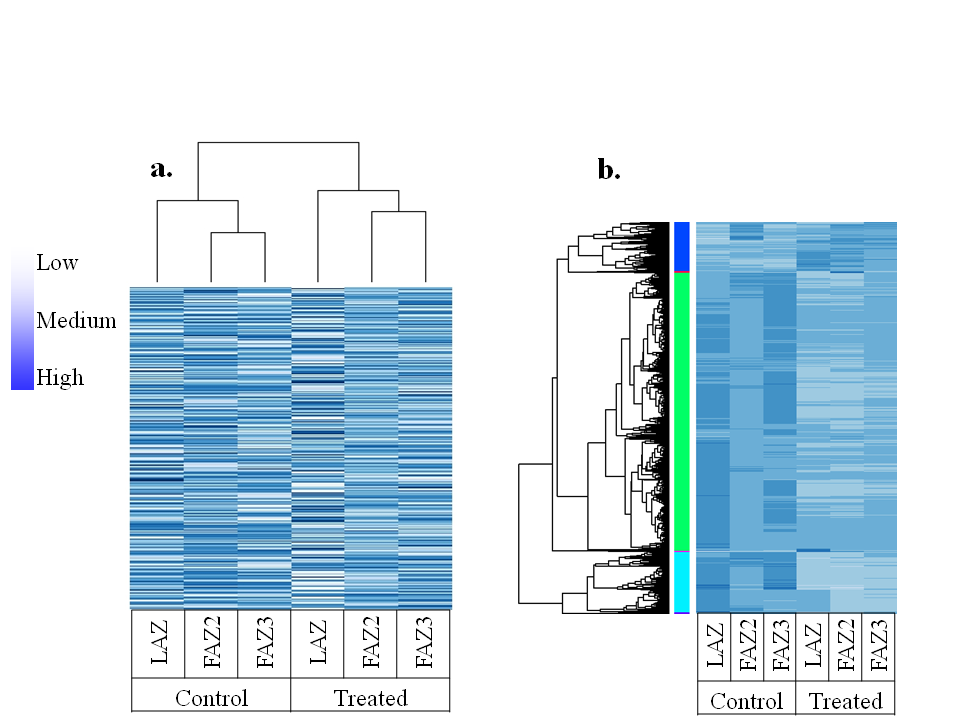

Supplement: Supplementary file 4 — Hierarchical clustering of all transcripts (a) and of differentially expressed transcripts (b) in the three AZs before and 5 days after ethephon treatment. Expression levels are indicated on an abundance scale of green to red. (TIFF 345 kb) [file 12870_2017_1035_MOESM4_ESM.tif]

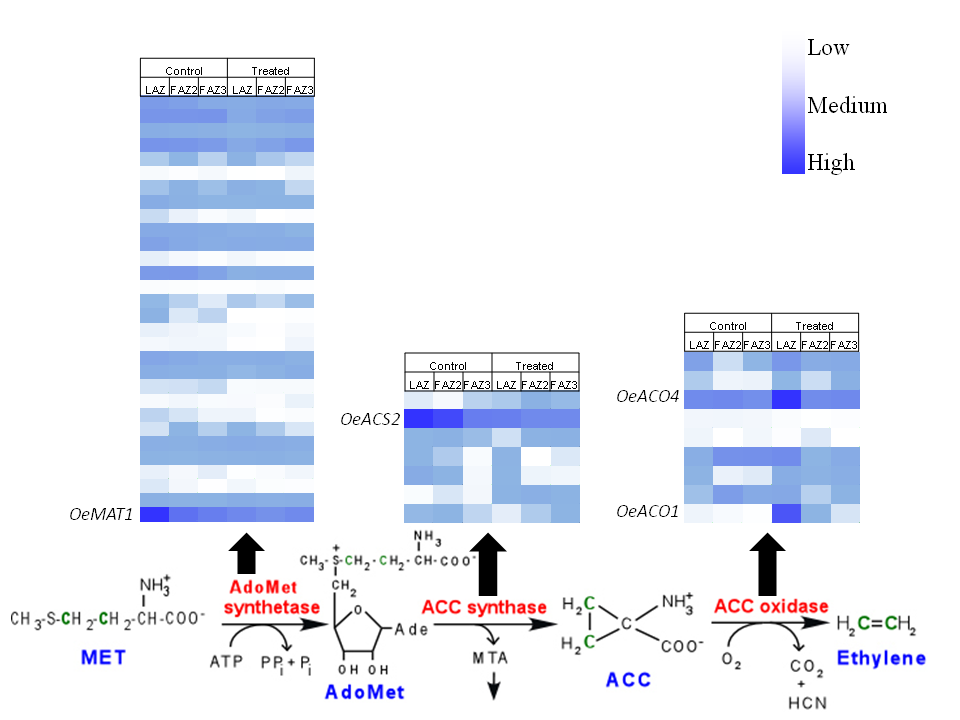

Supplement: Supplementary file 7 — Expression of genes involved in the ethylene biosynthesis pathway in the three AZs before and 5 days after ethephon treatment. Expression levels are indicated on an abundance scale of green to red. Only highly abundant transcripts appear with their gene names – S-Adenosylmethionine Synthase 1 (OeSAM1; OeMAT1), 1-Amino-Cyclopropane-1-Carboxylate Synthase 2 (OeACS2), 1-Amino-Cyclopropane-1-Carboxylate Oxidase 4 (OeACO4) and 1-Amino-Cyclopropane-1-Carboxylate Oxidase 1 (OeACO1). (TIFF 222 kb) [file 12870_2017_1035_MOESM7_ESM.tif]

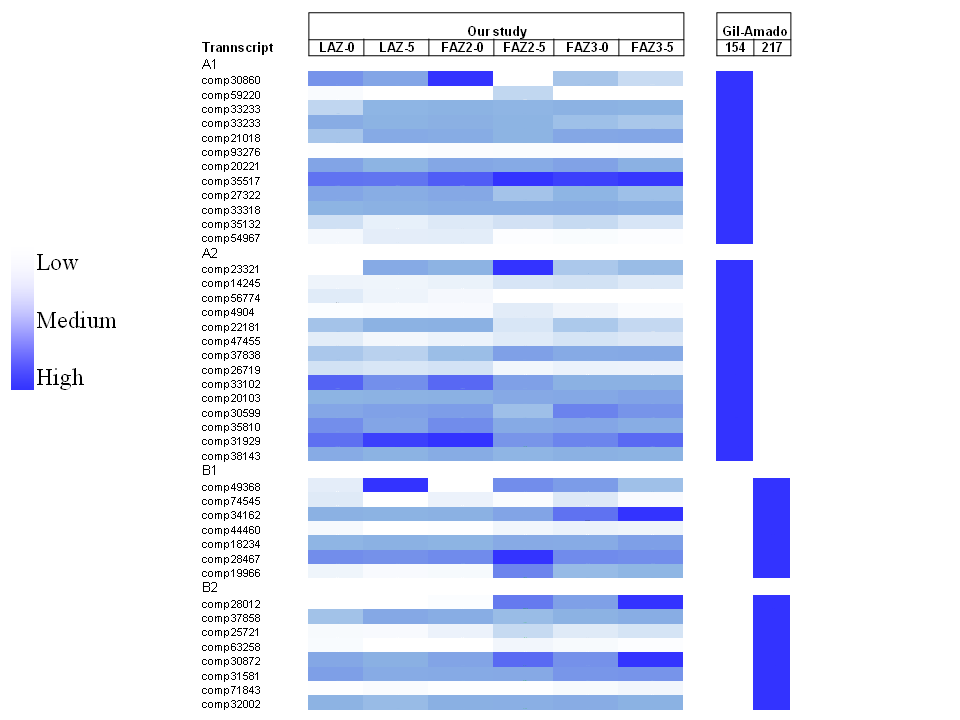

Supplement: Supplementary file 8 — Comparison between our results and those of Gil-Amado and Gomez-Jimenez [29]. The expression pattern of the highest ranked genes in each of the four clusters is shown. For each gene (rows) the expression levels in our study appear on the left whereas the expression levels of the same gene in an inactive AZ (154) and active AZ (217) according to Gil-Amado and Gomez-Jimenez appear on the right. (TIFF 172 kb) [file 12870_2017_1035_MOESM8_ESM.tif]

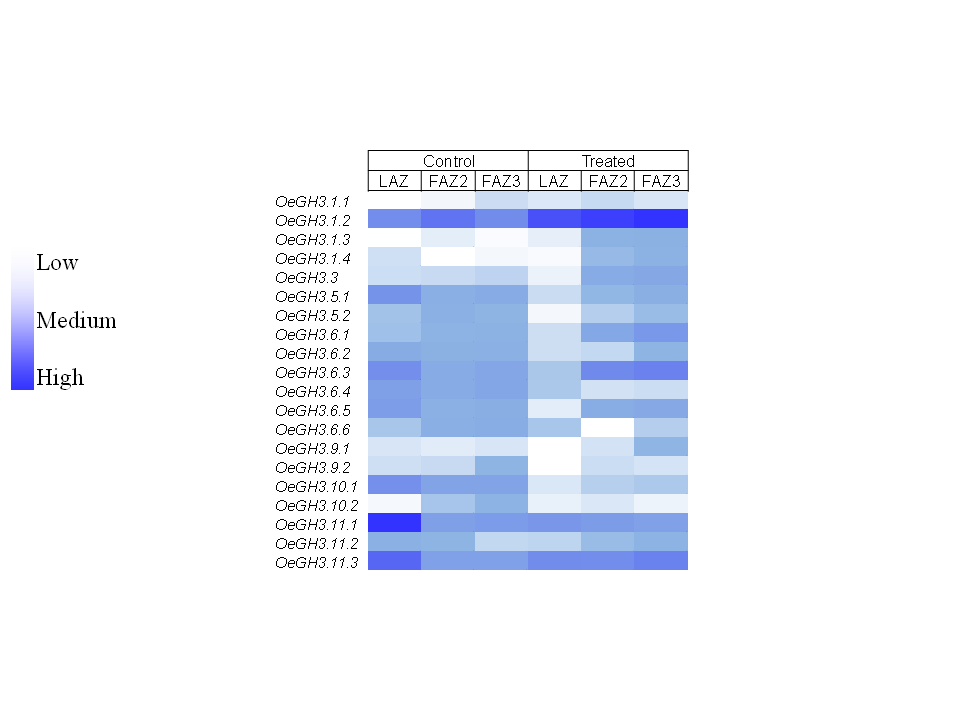

Supplement: Supplementary file 9 — Expression of genes belonging to the GH3 gene family in the three AZs before and 5 days after ethephon treatment. Expression level is presented in an abundance scale of green to red. The various columns represent the different tissues and treatments as indicated. (TIFF 116 kb) [file 12870_2017_1035_MOESM9_ESM.tif]
