# Supplementary material for: Ethephon induced oxidative stress in the olive leaf abscission zone enables development of a selective abscission compound
Source: BMC Plant Biol. 2017 May 16;17:87. doi: 10.1186/s12870-017-1035-1 (PMC5434568; doi:10.1186/s12870-017-1035-1)
Supplement: Supplementary file 3 — Deep sequencing and mapping statistics of the three AZs samples before and 5 days after ethephon treatment. (DOCX 14 kb) [file 12870_2017_1035_MOESM3_ESM.docx]

**Table S1:**

| Sample | Raw reads | Clean read | % mapping |
| --- | --- | --- | --- |
| FAZ2 - 5 | 34199751 | 31080113 | 81.78 |
| FAZ2 - 0 | 26638429 | 24081084 | 81.46 |
| FAZ3 - 5 | 50580223 | 45866476 | 83.6 |
| FAZ3 - 0 | 39062698 | 35279144 | 82.83 |
| LAZ - 5 | 28885660 | 26040384 | 79.61 |
| LAZ - 0 | 26716810 | 23891032 | 74.04 |
