# Supplementary material for: Ethephon induced oxidative stress in the olive leaf abscission zone enables development of a selective abscission compound
Source: BMC Plant Biol. 2017 May 16;17:87. doi: 10.1186/s12870-017-1035-1 (PMC5434568; doi:10.1186/s12870-017-1035-1)
Supplement: Supplementary file 6 — Relative abundances of all transcripts involved in biosynthesis of and in response to the plant hormones ethylene, auxin and ABA. Values are the average of logarithmic relative expression (5/0) of all three AZs (FPKM values). A statistical analysis was performed to determine if the average abundances of all transcripts in the three AZs are significantly higher 5 days after treatment as compared to untreated samples. The probability of each test is shown. (DOCX 29 kb) [file 12870_2017_1035_MOESM6_ESM.docx]

**Table S3:**

|  | Biosynthesis | | Response | |
| --- | --- | --- | --- | --- |
|  | LN(AZ5/0) | P | LN(AZ5/0) | P |
| Ethylene | 0.048 | 0.57 | 0.196 | **5.1X10^-7^** |
| Auxin | 0.606 | **2.69X10^-5^** | 0.275 | **2.8X10^-22^** |
| ABA | 0.236 | **0.0132** | 0.256 | **4.9X10^-31^** |
